# Cloning, Expression Analysis, and Functional Characterization of the NAC Transcription Factor *SlNAC2-like* from Tomato

**DOI:** 10.3390/plants15203213

**Published:** 2026-10-20

**Authors:** Lingzeng Lv, Lian Duan, Jingwei Liu, Haibo Li, Qin Shao, Na Chen

**Affiliations:** 1 School of Life Sciences and Environmental Resources, Yichun University, Yichun 336000, China; 19883696139@163.com (L.L.);; 2 Key Laboratory of Germplasm Innovation and Green High-Value Utilization for Specialty Crops in Western Jiangxi, Jiangxi Education Department, Yichun University, Yichun 336000, China

**Keywords:** tomato, NAC transcription factor, high-temperature tolerance, expression characteristic analysis, functional identification

## Abstract

To investigate the regulatory function of the tomato NAC transcription factor *SlNAC2-like* under high-temperature stress, the high-temperature-tolerant line Hm 2-2 and the high-temperature-sensitive line BY 1-2 were used as experimental materials. The *SlNAC2-like* gene was cloned from tomato leaves via reverse transcription PCR (RT-PCR), and its encoded protein was characterized using bioinformatics tools, phylogenetic analysis, and expression profiling. Subcellular localization and yeast self-activation assays were performed to determine protein localization and transcriptional activity. Virus-induced gene silencing (VIGS) was employed to generate *SlNAC2-like*-silenced plants, and the activities of superoxide dismutase (SOD), peroxidase (POD), and catalase (CAT), as well as proline (Pro) and malondialdehyde (MDA) contents, were measured under high-temperature stress using spectrophotometry. The results showed that the *SlNAC2-like* cDNA fragment was 1044 bp in length, with an 876 bp open reading frame encoding 291 amino acids containing a highly conserved NAM domain. The protein was localized in the nucleus and exhibited transcriptional activation activity. Quantitative real-time PCR (qRT-PCR) analysis revealed that *SlNAC2-like* expression was tissue-specific and induced by high temperature (42 °C), abscisic acid (ABA), salicylic acid (SA), and methyl jasmonate (MeJA). Silencing of *SlNAC2-like* compromised tomato high-temperature tolerance, as evidenced by reduced SOD, POD, and CAT activities, decreased Pro content, and a marked increase in MDA content, along with significant downregulation of genes involved in ABA, SA, and jasmonic acid (JA) signaling pathways. Collectively, these findings suggest that *SlNAC2-like* acts as a positive regulator of tomato tolerance to high-temperature stress and that its function is associated with antioxidant defense and the ABA, SA, and JA signaling pathways. However, the present study does not establish how SlNAC2-like regulates ROS homeostasis or hormone signaling, and identification of its direct target genes and interacting partners requires further experimental validation. This study provides valuable genetic resources and a theoretical basis for molecular breeding of stress-resistant tomato varieties.

## 1. Introduction

Tomato (*Solanum lycopersicum* L.) is an important vegetable crop of the Solanaceae family, widely cultivated across the globe. It ranks among the most consumed agricultural products worldwide and serves as a significant source of vitamins and bioactive compounds in the human diet [1]. However, tomato plants are highly sensitive to high-temperature stress; temperatures exceeding 35 °C can severely disrupt normal growth and development, leading to reduced yield and deteriorated fruit quality [2]. With the continuous rise in global temperatures, high-temperature stress poses a major threat to crop productivity [3]. Plant high-temperature tolerance is influenced by environmental factors, management practices, and genotype [4]. Therefore, the most fundamental approach to addressing this challenge is to identify key high-temperature-responsive genes and develop high-temperature-tolerant varieties through molecular breeding.

Transcription factors are involved in regulating nearly all aspects of plant growth and development and can construct sophisticated regulatory networks to enhance plant resistance to both abiotic and biotic stresses [5]. The NAC family represents one of the most important and plant-specific transcription factor families, participating extensively in growth, development, and responses to various environmental stresses [6,7,8,9]. In recent years, considerable efforts have been devoted to the identification and functional characterization of high-temperature-tolerance genes in plants. For instance, Bo et al. [10] reported that overexpression of the NAC transcription factor *PtNAC66* from *Pinellia ternata* enhanced heat tolerance in *Arabidopsis* by binding to and repressing the expression of *CYP707A3*, *MYB102*, and *NAC055*. Wu et al. [11] showed that *LlNAC014* from lily (*Lilium longiflorum*) perceives heat signals through its C-terminal transmembrane domain and translocates into the nucleus, where it directly activates the DREB2-HSFA3 module to trigger heat shock responses, thereby improving thermotolerance. In contrast, Yang et al. [12] found that lettuce *LsNAC46* negatively regulates heat tolerance. Meng et al. [13] demonstrated that tomato *SlNAC72* negatively modulates heat tolerance by directly suppressing the expression of key jasmonic acid (JA) biosynthesis genes, *SlLOX10*, *SlAOS1*, and *SlAOC*. Zhao et al. [14] reported that *DcNAC41* from carnation is significantly induced by high temperature, and its overexpression markedly enhances heat tolerance in transgenic *Arabidopsis* by improving photosynthetic capacity and reactive oxygen species (ROS) scavenging ability. Wang et al. [15] revealed that *OsNAC006*, a transcription factor in rice (*Oryza sativa* L.), is induced by multiple abiotic stresses and phytohormones, and CRISPR-Cas9 knockout mutants exhibit drought- and heat-sensitive phenotypes; *OsNAC006* plays a critical role in stress tolerance by regulating genes involved in stimulus response, oxidoreductase activity, and membrane-associated pathways. Collectively, these findings highlight the pivotal role of NAC transcription factors in the regulation of high-temperature stress responses in plants.

To date, relatively few studies have addressed the involvement of NAC transcription factors in the regulation of high-temperature stress responses in tomato. In our previous work, we compared the transcriptomes of a high-temperature-tolerant tomato line (Hm 2-2) and a high-temperature-sensitive line (BY 1-2) under high-temperature treatment and identified a NAC transcription factor gene, *SlNAC2-like* (Solyc11g017470), that was differentially expressed between the two lines and markedly induced by high-temperature stress. *SlNAC2-like* was selected for further study for the following reasons. First, it belongs to the plant-specific NAC family, members of which play key roles in abiotic stress responses, including thermotolerance. Second, its expression was strongly and rapidly induced by high temperature in the high-temperature-tolerant line, suggesting a potential role in high-temperature stress adaptation. Third, its expression differed between the high-temperature-tolerant and high-temperature-sensitive lines, indicating that it may contribute to the genotypic differences in high-temperature tolerance. Fourth, although several tomato NAC genes have been characterized, the function of *SlNAC2-like* in high-temperature tolerance has not been reported, and it appears distinct from the previously described negative regulator *SlNAC72*. In this study, we cloned this gene using reverse transcription PCR (RT-PCR) and performed bioinformatics analyses. Furthermore, we investigated the expression patterns and functional roles of *SlNAC2-like* under high-temperature stress using quantitative real-time PCR (qRT-PCR) and virus-induced gene silencing (VIGS). VIGS was chosen because it allows rapid, transient knockdown of target genes in tomato without the need for stable transformation, which is particularly useful for the initial functional characterization of candidate genes. Unlike stable knockout or overexpression lines, however, VIGS induces only partial silencing and acts transiently; therefore, the results should be interpreted as evidence that *SlNAC2-like* is involved in, rather than as direct proof that it is necessary and sufficient for, tomato high-temperature tolerance. In addition, virus-mediated silencing may have background effects, which is why TRV::Empty plants were used as controls throughout this study. Our findings are expected to provide a theoretical basis for a deeper understanding of the regulatory mechanisms through which NAC transcription factors mediate tomato responses to high-temperature stress.

## 2. Results

### 2.1. Cloning and Structural Analysis of Tomato SlNAC2-like

**Figure 1:**
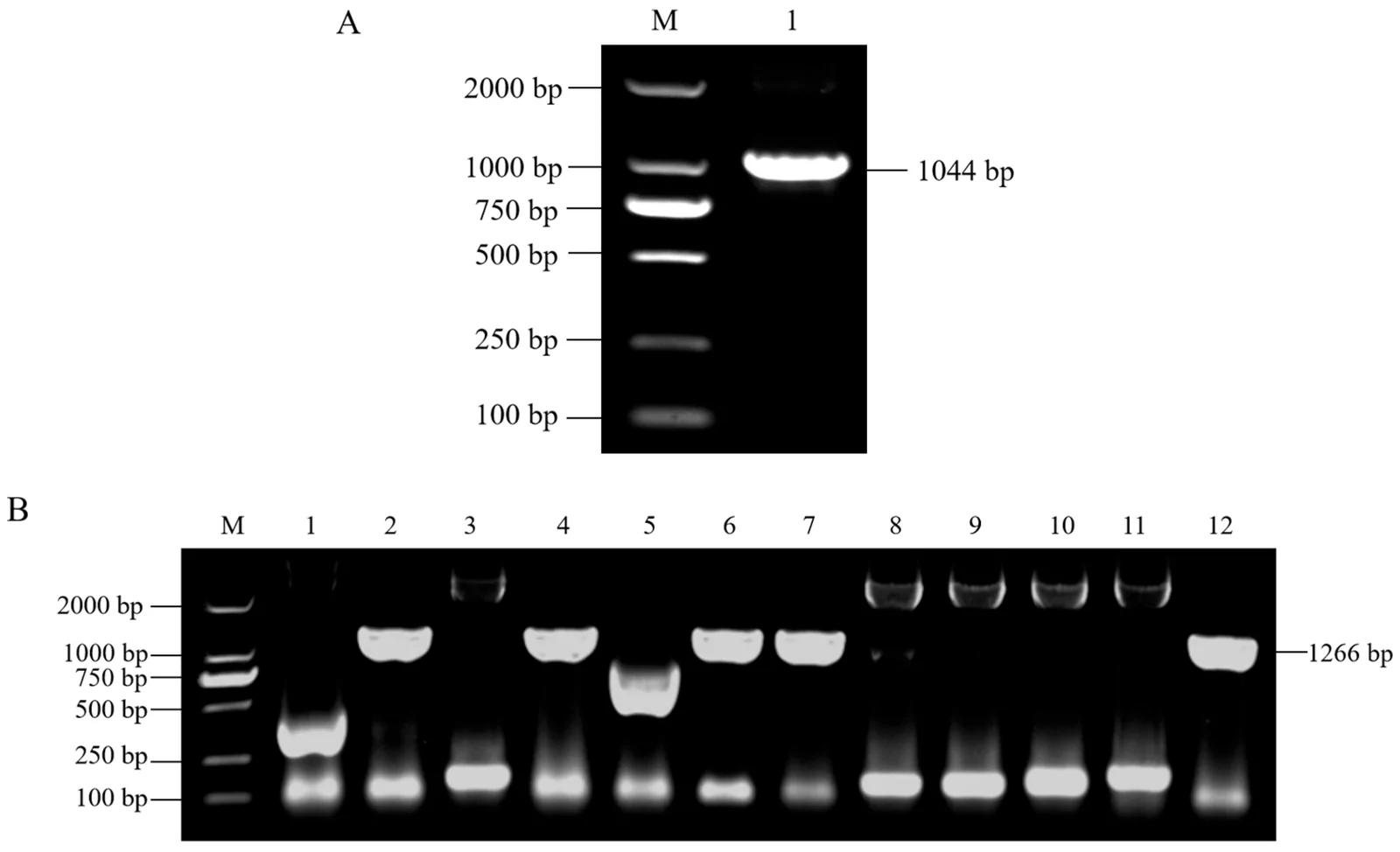
Tomato *SlNAC2-like* gene. (**A**) M: Mark DL 2000; 1: *SlNAC2-like* gene. (**B**) M: Mark DL 2000; 2, 4, 6–7, 12: positive clone; 1, 3, 5, 8~11: negative clone.

**Figure 2:**
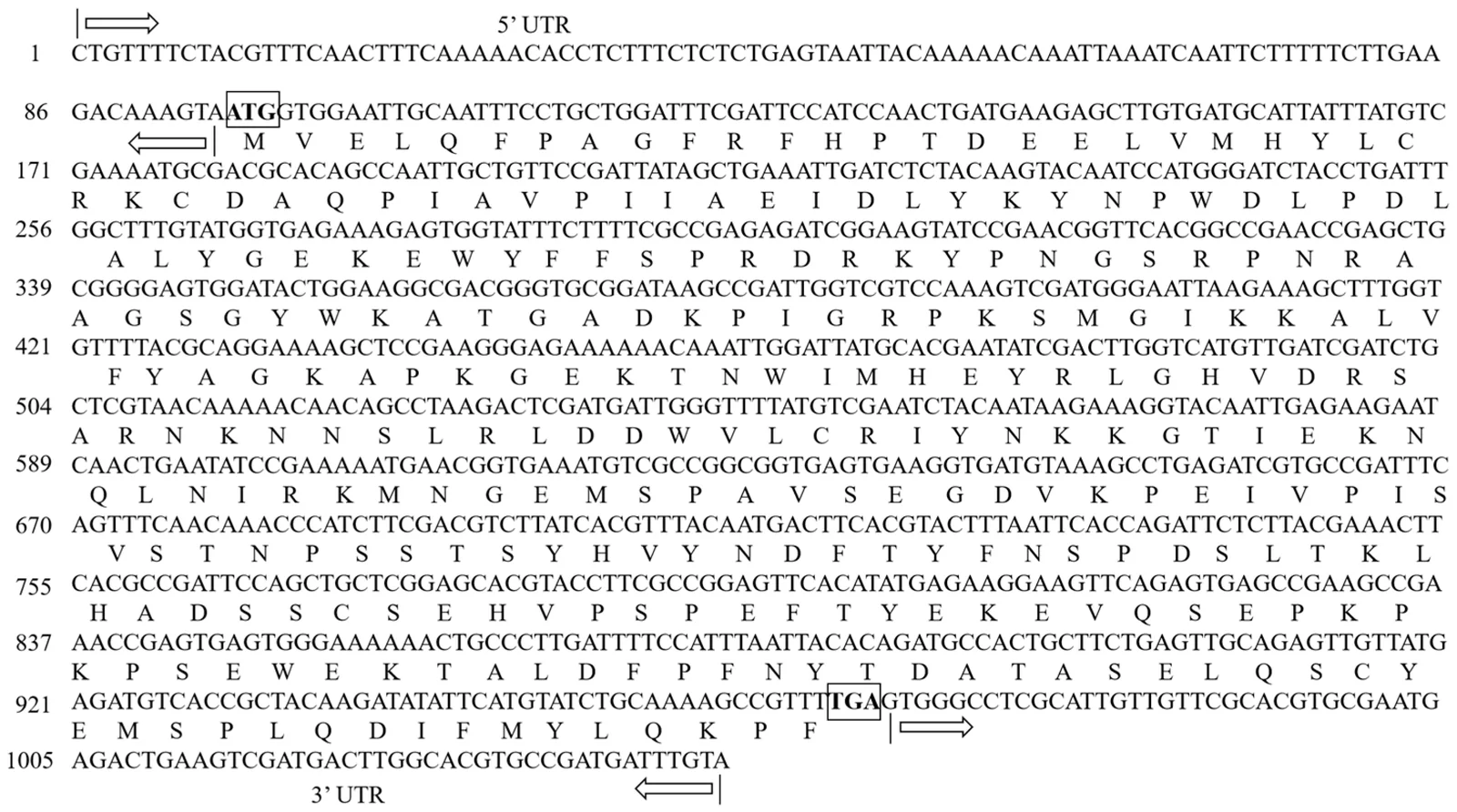
*SlNAC2-like* cDNA sequence and the predicted open reading frame.

**Figure 3:**
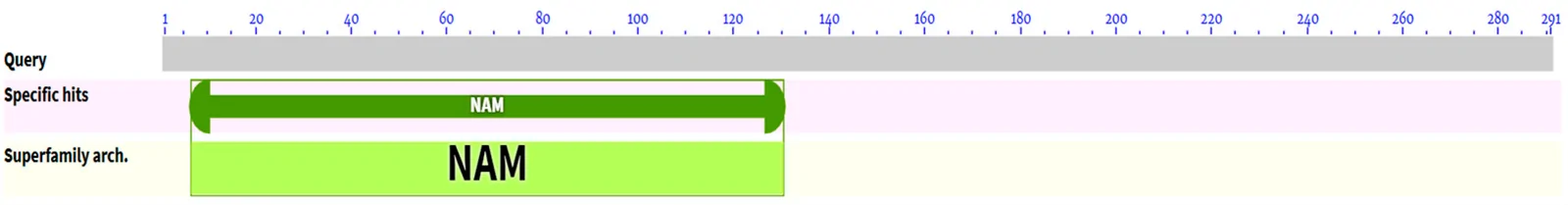
Conserved region of SlNAC2-like protein.

PCR amplification was performed using cDNA from leaves of the tomato line BY 1-2 as a template. Agarose gel electrophoresis revealed a single band of the expected size corresponding to the target fragment (Figure 1A). The purified PCR product was ligated into the pMD19-T vector (Figure 1B). The sequencing results showed that the full-length cDNA of *SlNAC2-like* was 1044 bp, containing an 876 bp open reading frame (ORF) (Figure 2) encoding 291 amino acids (positions 95–970 bp). Conserved domain analysis (Figure 3) revealed that the protein contains a highly conserved NAM domain (positions 7–130 aa), indicating that it belongs to the NAC transcription factor family.

### 2.2. Bioinformatics Analysis of Tomato SlNAC2-like

**Figure 4:**
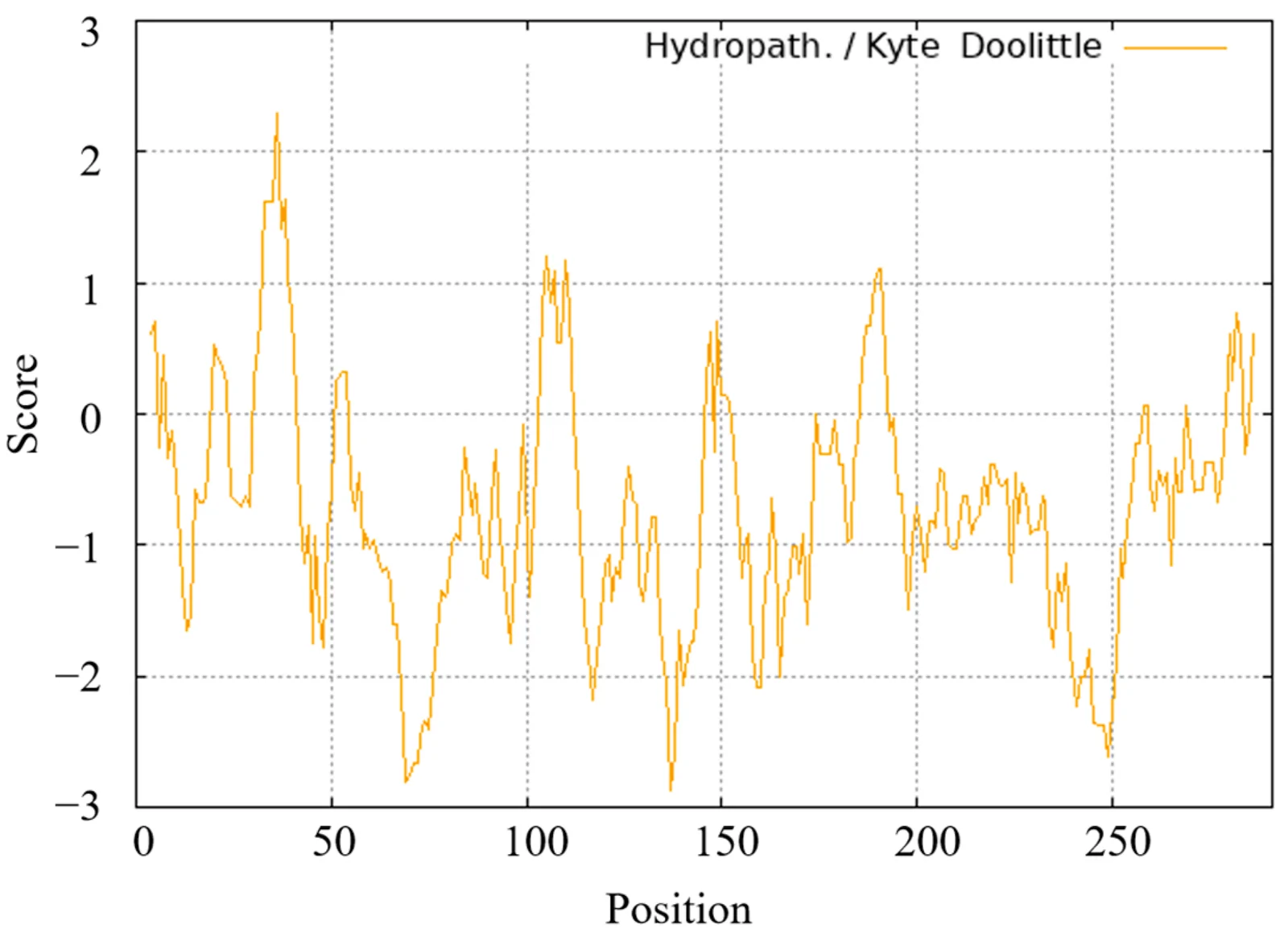
Analysis of the hydrophobicity of SlNAC2-like protein.

**Figure 5:**
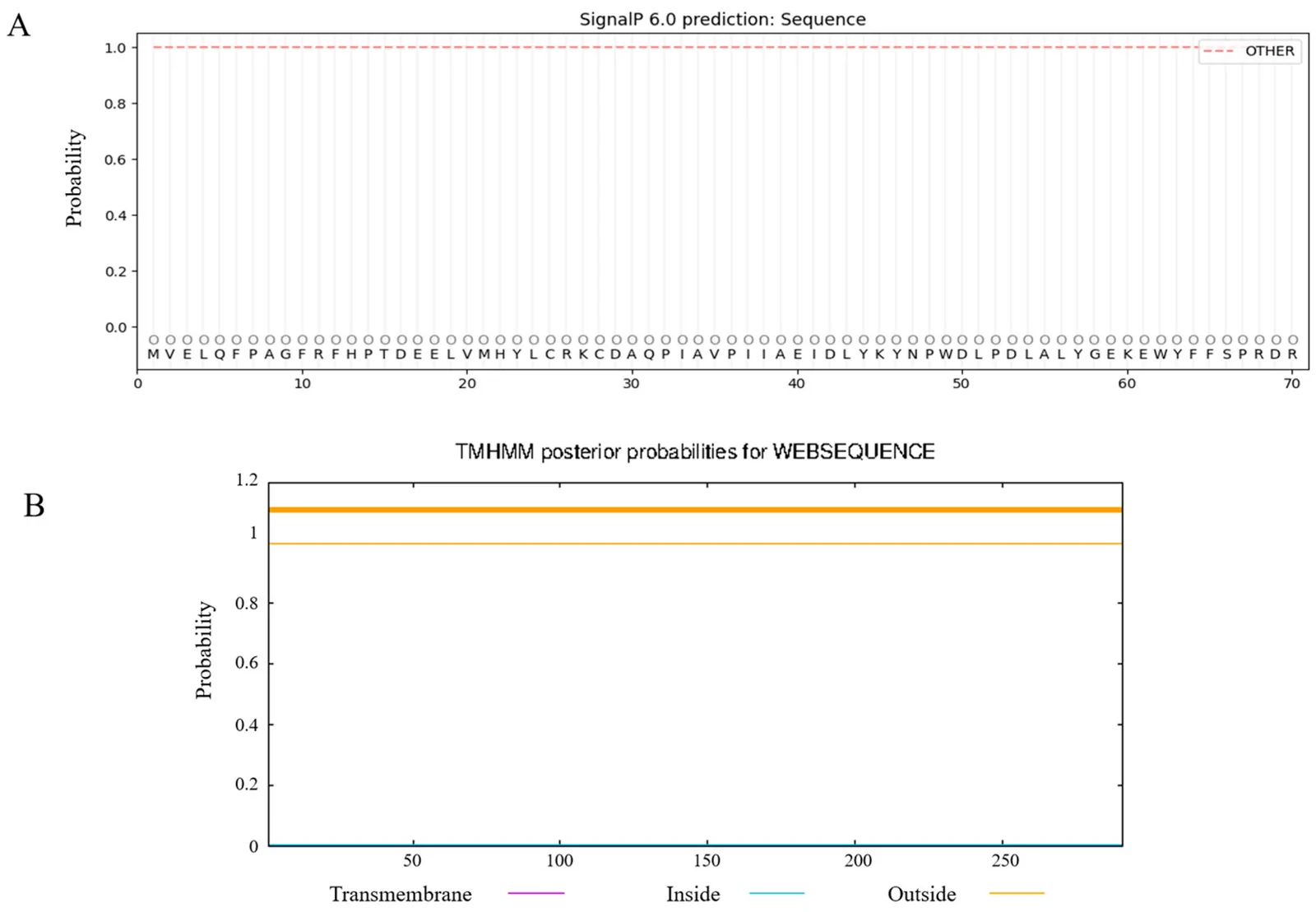
Analysis of the signal peptide and transmembrane structure of the SlNAC2-like protein. (**A**) Prediction of the signal peptide of the SlNAC2-like protein. (**B**) Prediction of the transmembrane structure of SlNAC2-like protein.

**Figure 6:**
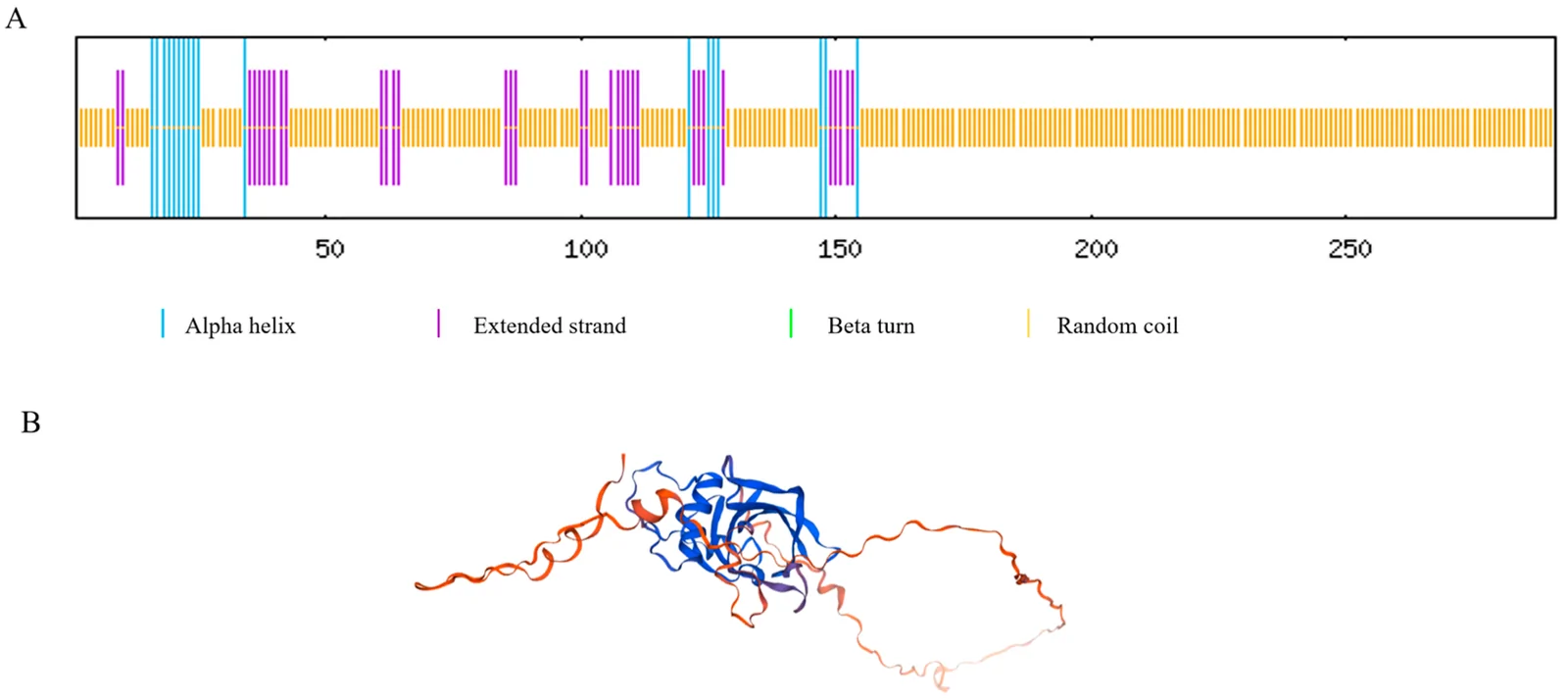
Prediction of the secondary and tertiary structures of SlNAC2-like protein. (**A**) Secondary structure of SlNAC2-like protein. (**B**) Tertiary structure of SlNAC2-like protein.

Online program analysis showed that the SlNAC2-like protein has a molecular formula of C_1507_H_2280_N_396_O_443_S_13_, a molecular weight of 33,449.85 Da, and an isoelectric point of 6.67. It contains 38 negatively charged residues (Asp + Glu) and 37 positively charged residues (Arg + Lys), with an aliphatic index of 62.03, an instability index of 55.37, and a grand average of hydropathicity (GRAVY) of −0.699, indicating hydrophilicity (Figure 4). SignalP 6.0 and TMHMM Server v. 2.0 predicted that the protein lacks a signal peptide and transmembrane domains (Figure 5). Secondary structure analysis revealed that random coil, α-helix, extended strand, and β-turn accounted for 82.13%, 6.19%, 11.68%, and 0.00%, respectively (Figure 6A), with random coil being the predominant form. The three-dimensional structural modeling results were consistent with the secondary structure prediction (Figure 6B).

**Figure 7:**
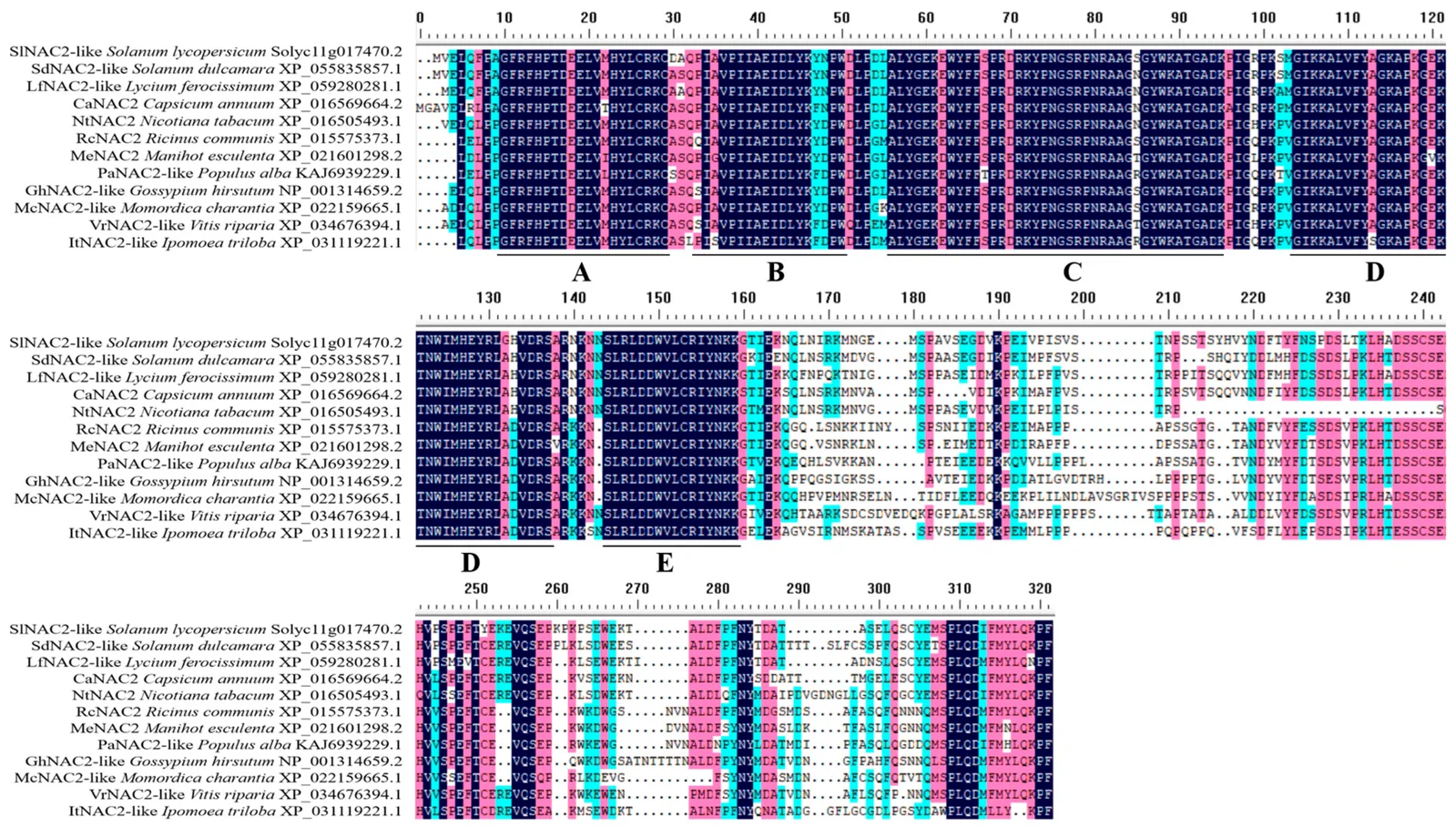
Multiple sequence alignment of SlNAC2-like protein in tomato and homologous proteins from other species. (**A**–**E**): NAC subdomain.

**Figure 8:**
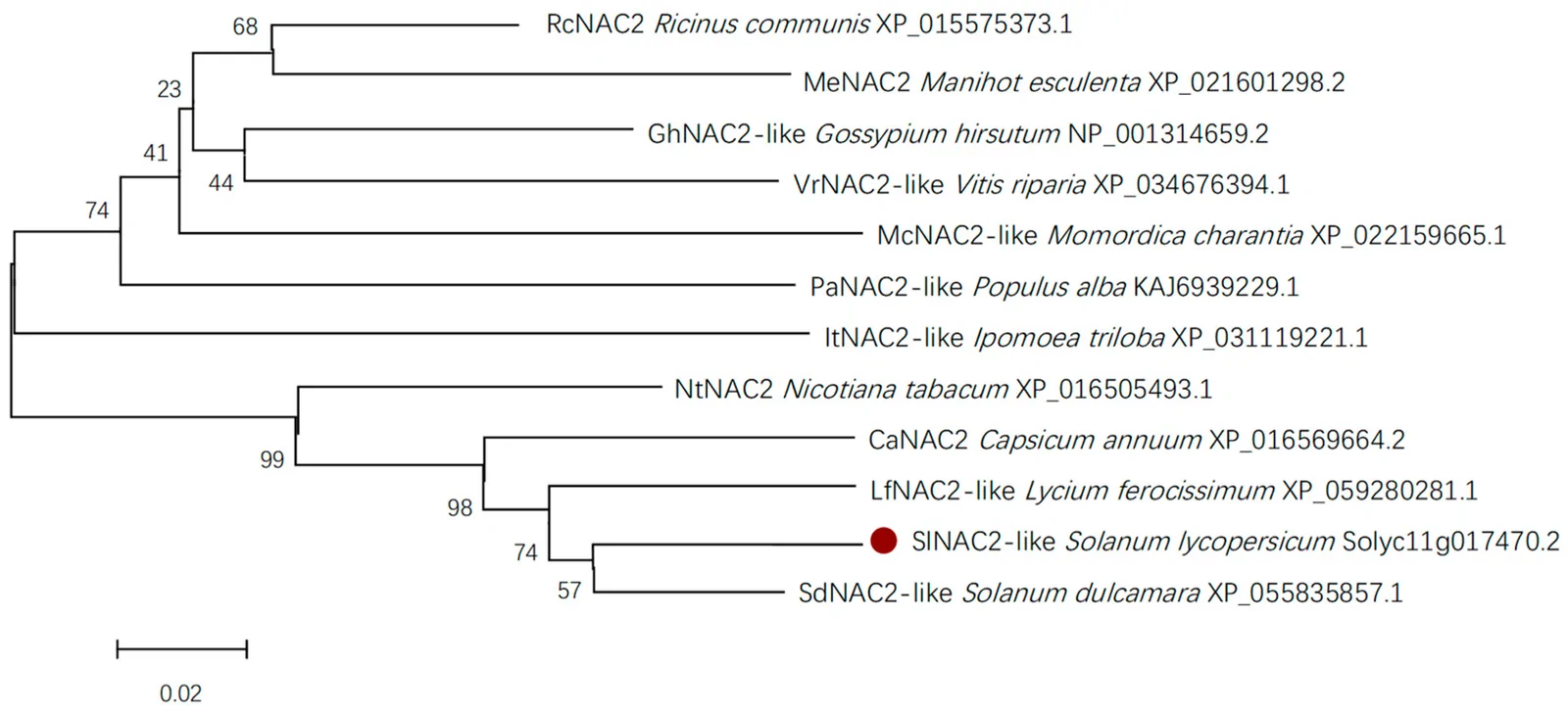
Phylogenetic tree of SlNAC2-like and its homologous proteins.

Multiple sequence alignment of NAC protein sequences from tomato and 11 other species, including *Solanum dulcamara*, *Lycium ferocissimum*, *Capsicum annuum*, and others, revealed that the N-terminal amino acid sequences of these NAC proteins are highly conserved and could be divided into five subdomains (A–E) (Figure 7). Phylogenetic tree analysis indicated that SlNAC2-like is most closely related to SdNAC2-like from *S. dulcamara* and most distantly related to RcNAC2 from *Ricinus communis* (Figure 8).

### 2.3. Expression Pattern Analysis of SlNAC2-like

**Figure 9:**
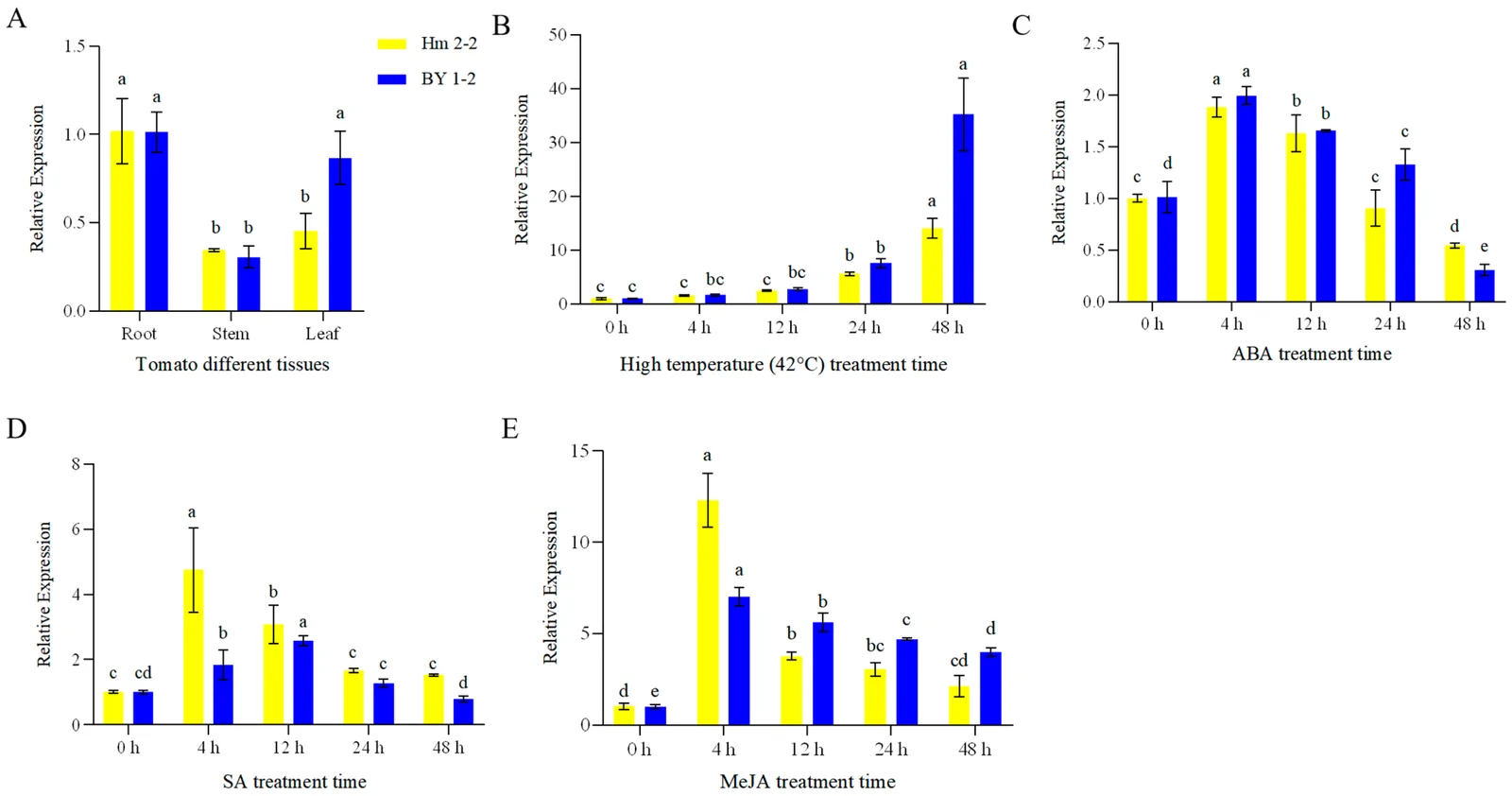
Analysis of the expression characteristics of tomato *SlNAC2-like*. (**A**) Expression of *SlNAC2-like* in different tissues of tomato. (**B**) Effects of high-temperature treatment on *SlNAC2-like* expression. (**C**) Effects of exogenous hormone ABA treatment on *SlNAC2-like* expression. (**D**) Effects of exogenous hormone SA treatment on *SlNAC2-like* expression. (**E**) Effects of exogenous hormone MeJA treatment on *SlNAC2-like* expression. *n* = 3 biological replicates. For each tomato line (Hm 2-2 and BY 1-2), the different time points (or tissues) were compared together by one-way ANOVA followed by Tukey’s HSD test. Different lowercase letters indicate significant differences (*p* < 0.05).

qRT-PCR results showed that the expression level of *SlNAC2-like* in the roots of Hm 2-2 was significantly higher than that in stems and leaves, while in BY 1-2, the expression levels in both roots and leaves were significantly higher than that in stems (Figure 9A). This indicates that its expression in tomato is tissue-specific. After high-temperature treatment, the expression level of *SlNAC2-like* showed an upward trend (Figure 9B) and was positively correlated with treatment time, suggesting that high temperature can significantly induce the expression of this gene. Following treatment with three hormones, abscisic acid (ABA), salicylic acid (SA), and methyl jasmonate (MeJA), the expression levels of *SlNAC2-like* in both materials exhibited a trend of first increasing and then decreasing (Figure 9C–E). These results suggest that *SlNAC2-like* is responsive to ABA, SA, and MeJA and that it may be associated with these hormone signaling pathways in the tomato response to high-temperature stress.

### 2.4. Subcellular Localization and Yeast Autoactivation Assay

**Figure 10:**
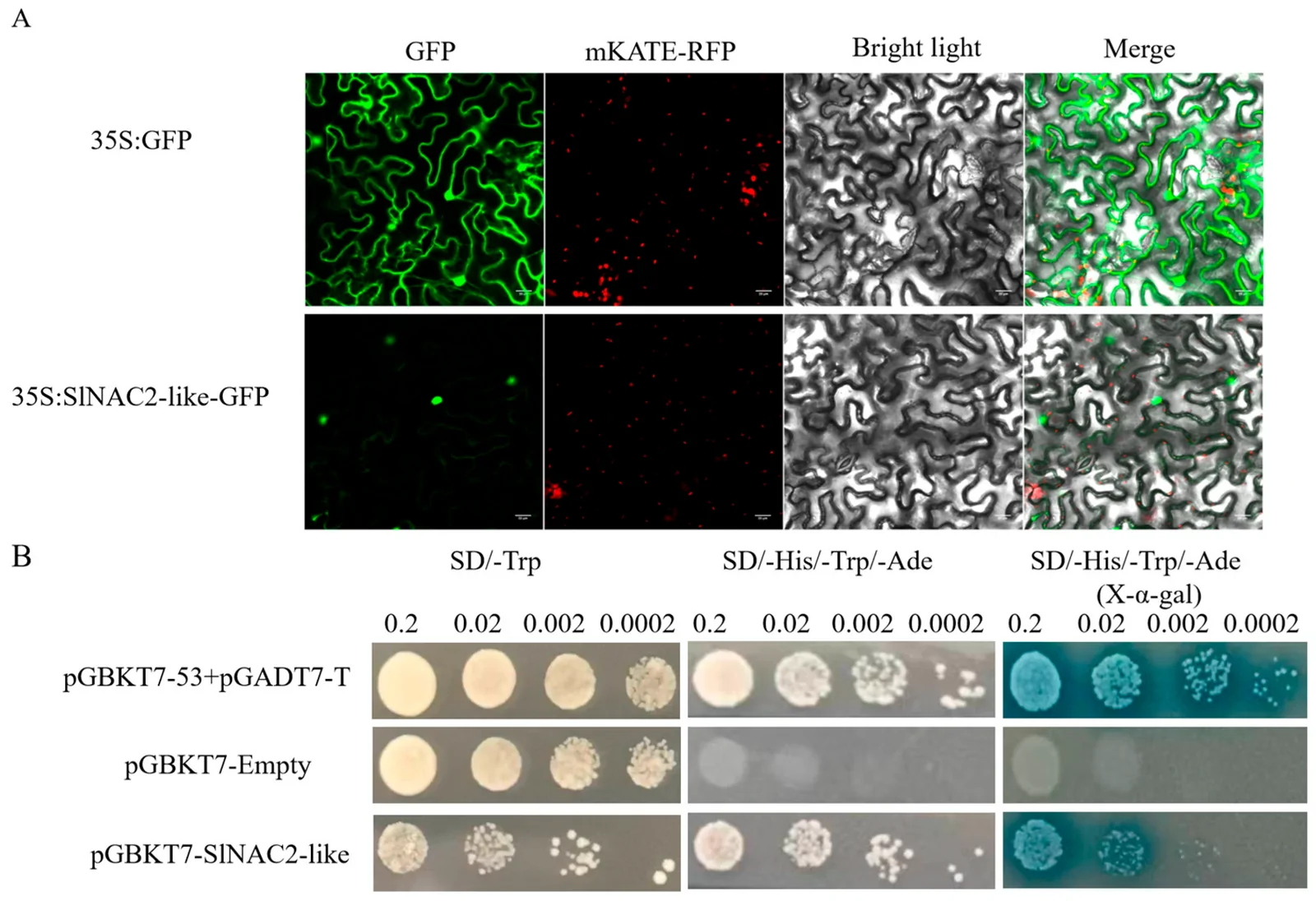
Subcellular localization and transcriptional activation of SlNAC2-like. (**A**) Subcellular localization of SlNAC2-like in tobacco epidermal cells. Scale bar = 20 μm. (**B**) Transcriptional activation of SlNAC2-like in yeast.

The subcellular localization results are shown in Figure 10A. The GFP signal derived from the 35S:SlNAC2-like-GFP recombinant vector was mainly detected in the nuclear region, whereas the GFP signal from the control (35S:GFP) was detected in both the cytoplasm and the nucleus, indicating that SlNAC2-like is a nuclear protein.

The yeast autoactivation assay results are shown in Figure 10B. All yeast cells grew normally on SD/-Trp medium. Furthermore, only the cells carrying the positive control combination (pGADT7-T + pGBKT7-53) and those carrying the pGBKT7-SlNAC2-like recombinant vector grew and turned blue on SD/-His/-Trp/-Ade medium or SD/-His/-Trp/-Ade medium containing X-α-gal. In contrast, yeast cells carrying the pGBKT7 empty vector failed to grow on SD/-His/-Trp/-Ade medium. These results indicate that SlNAC2-like possesses transcriptional activation activity.

### 2.5. Silencing SlNAC2-like Reduces Tomato Plant Resistance to High Temperature

**Figure 11:**
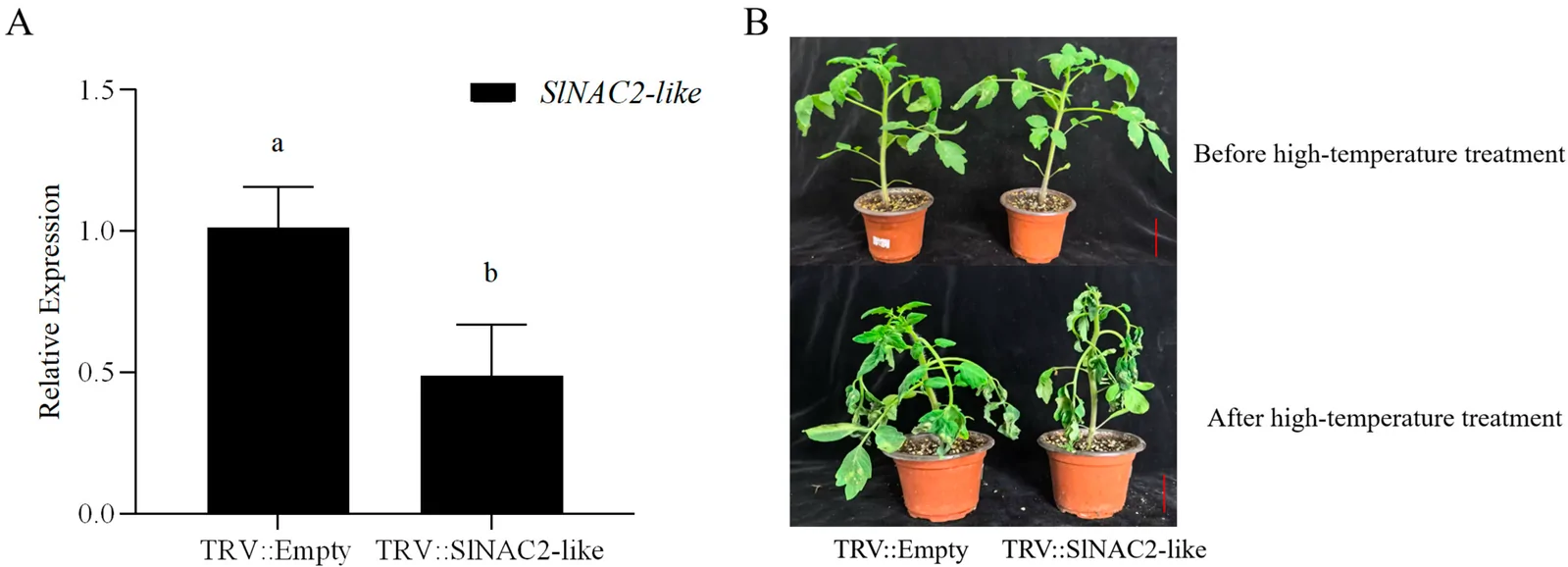
*SlNAC2-like* positively regulates the resistance of tomatoes to high temperatures. (**A**) The relative expression level of *SlNAC2-like* in TRV-mediated *SlNAC2-like* silencing tomato plants. Leaf samples were collected at 2 days post-infiltration. *n* = 3 biological replicates. Data were analyzed by one-way ANOVA followed by Tukey’s HSD test. Different lowercase letters indicate significant differences (*p* < 0.05). (**B**): Phenotypic characteristics of tomato plants with TRV-mediated *SlNAC2-like* gene silencing before and after high-temperature treatment. TRV::Empty: control group, inoculated with empty virus vector; TRV::SlNAC2-like: VIGS treatment group, transfected with *SlNAC2-like* recombinant virus vector. Scale bar = 5 cm.

Functional analysis results showed that after silencing *SlNAC2-like* in tomato Hm 2-2, the transcript level of *SlNAC2-like* in TRV::SlNAC2-like plants was reduced to approximately 48.16% of that in TRV::Empty plants at 2 days post-infiltration (Figure 11A), confirming that silencing was established within 48 h after agroinfiltration and that TRV::SlNAC2-like infection effectively silenced *SlNAC2-like* in Hm 2-2. After high-temperature treatment of the silenced plants, it was observed that 1–2 leaves of the TRV::Empty-infected plants showed wilting, whereas nearly all leaves of the TRV::SlNAC2-like-infected plants exhibited obvious wilting symptoms after the same treatment (Figure 11B). This phenotypic difference was observed consistently across the three biological replicates and is described here as a qualitative observation. These results indicate that silencing *SlNAC2-like* reduces the resistance of tomato plants to high temperature and that *SlNAC2-like* plays a positive regulatory role in this process.

### 2.6. Effects of Silencing SlNAC2-like on Antioxidant Enzyme Activities and Expression of Signaling-Related Genes

**Figure 12:**
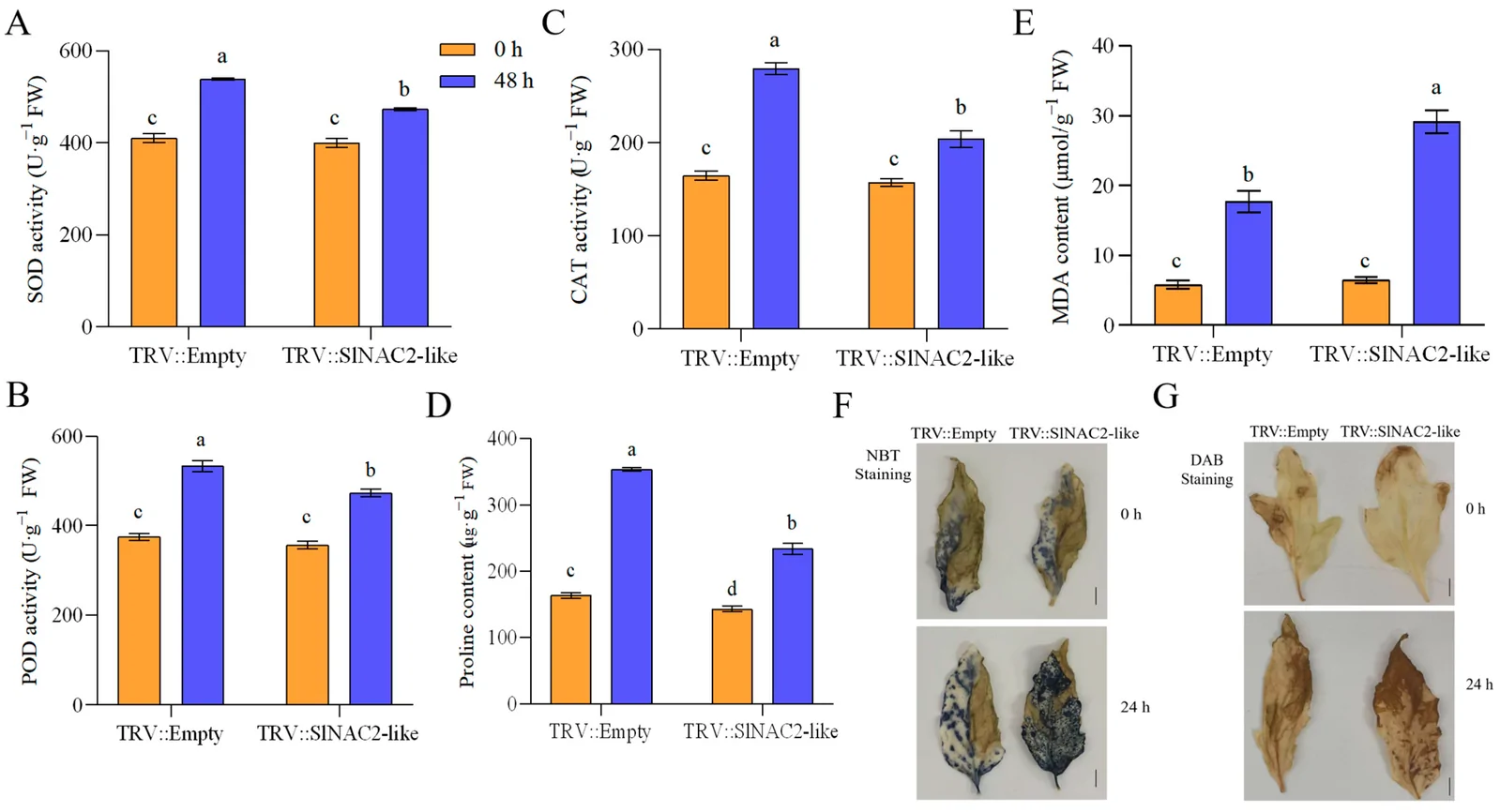
The influence of silencing *SlNAC2-like* on antioxidant enzyme activity. (**A**–**C**) Activities of superoxide dismutase (SOD), peroxidase (POD), and catalase (CAT). (**D**,**E**) Contents of proline (Pro) and malondialdehyde (MDA). (**F**,**G**) Nitroblue tetrazolium (NBT) and 3,3′-diaminobenzidine (DAB) staining. *n* = 3 biological replicates. The four groups (TRV::Empty-0 h, TRV::Empty-48 h, TRV::SlNAC2-like-0 h, and TRV::SlNAC2-like-48 h) were analyzed together by one-way ANOVA followed by Tukey’s HSD test. Different lowercase letters indicate significant differences (*p* < 0.05) (**A**–**E**). Scale bar = 0.3 cm.

The results of antioxidant enzyme activity analysis are shown in Figure 12. Under high-temperature treatment, the activities of superoxide dismutase (SOD), peroxidase (POD), and catalase (CAT), as well as the proline (Pro) content, in SlNAC2-like-silenced plants were significantly lower than those in control plants, whereas the malondialdehyde (MDA) content was significantly increased (Figure 12A–E). Nitroblue tetrazolium (NBT) and 3,3′-diaminobenzidine (DAB) staining analyses showed that hydrogen peroxide (H_2_O_2_) and superoxide anion (O_2_^–^) accumulation appeared more pronounced in *SlNAC2*-like-silenced plants than in control plants (Figure 12F,G).

### 2.7. Effects of Silencing SlNAC2-like on the Expression of Hormone Signaling-Related Genes

**Figure 13:**
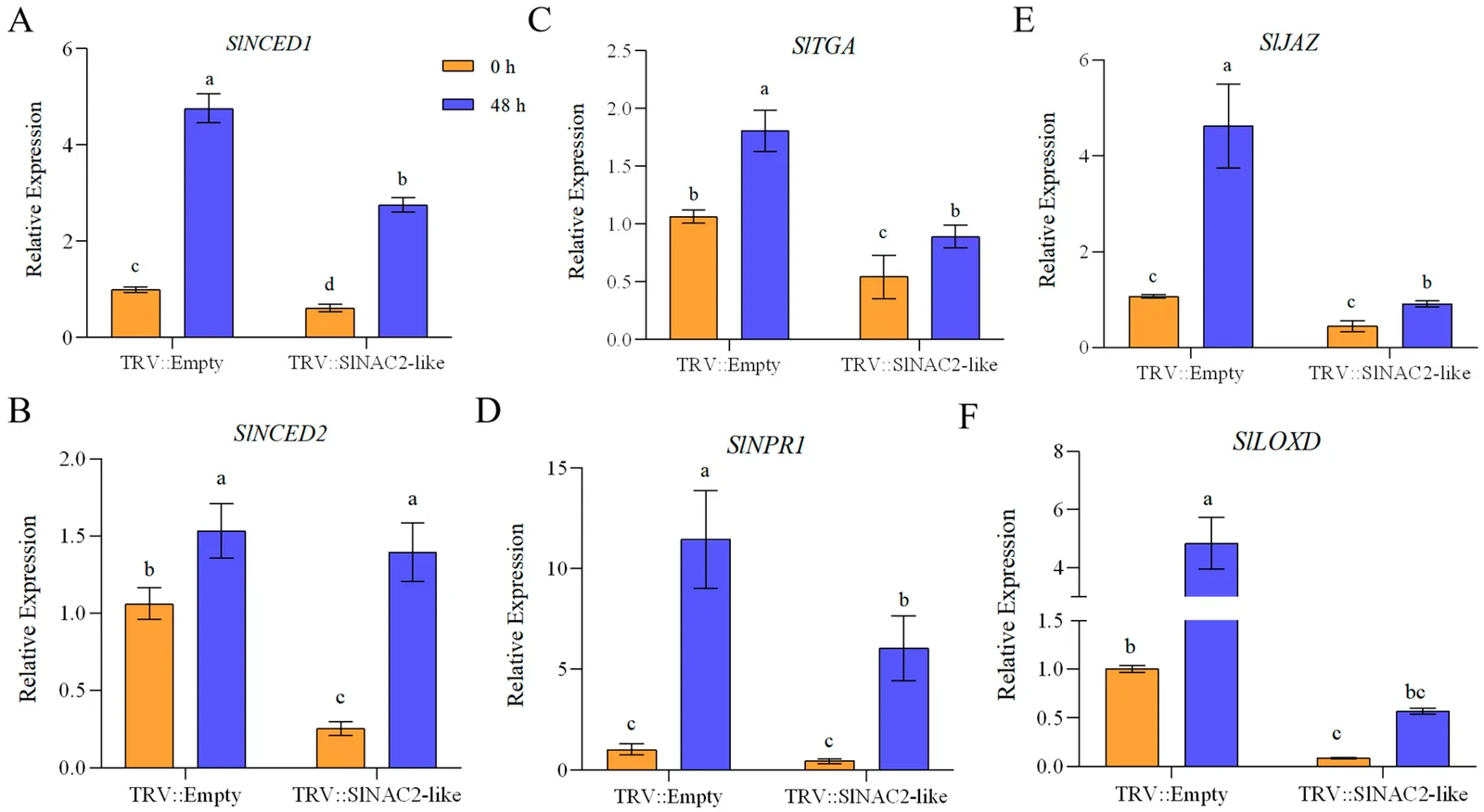
The influence of SlNAC2-like on genes related to hormone signaling pathways. (**A**,**B**) Genes related to the ABA signaling pathway. (**C**,**D**) Genes related to the SA signaling pathway. (**E**,**F**) Genes related to the JA signaling pathway. *n* = 3 biological replicates. The four groups (TRV::Empty-0 h, TRV::Empty-48 h, TRV::SlNAC2-like-0 h, and TRV::SlNAC2-like-48 h) were analyzed together by one-way ANOVA followed by Tukey’s HSD test. Different lowercase letters indicate significant differences (*p* < 0.05).

The expression of hormone signaling-related genes was measured by qRT-PCR, and the results are shown in Figure 13. After high-temperature treatment, the expression levels of genes involved in hormone signaling pathways were significantly upregulated. Compared with the control plants (TRV::Empty), the expression levels of ABA pathway-related genes (*SlNCED1*, Solyc07g056570 and *SlNCED2*, Solyc08g016720), SA pathway-related genes (*SlTGA*, Solyc11g064950 and *SlNPR1*, Solyc07g040690), and JA pathway-related genes (*SlJAZ*, Solyc12g009220 and *SlLOXD*, Solyc03g122340) were significantly decreased in *SlNAC2-like*-silenced plants (TRV::SlNAC2-like). These results indicate an association between *SlNAC2-like* and these hormone signaling pathways, rather than direct transcriptional regulation.

## 3. Discussion

High temperature is one of the major abiotic stress factors negatively affecting plant growth and reproduction worldwide, and its impact on crop yield and quality has become increasingly significant with climate change [16]. Previous studies have confirmed that NAC transcription factors play important roles in plant resistance to high-temperature stress, but relatively few studies have focused on the involvement of NAC transcription factors in high-temperature regulation in tomato. In this study, we cloned the NAC transcription factor *SlNAC2-like* from tomato and characterized its expression pattern and functional roles, which is of great significance for exploring the regulatory function of NAC transcription factors in tomato responses to high-temperature stress. Protein analysis of SlNAC2-like revealed that it is a hydrophilic protein, consistent with the findings of Yang et al. [17]. The SlNAC2-like protein was mainly composed of random coils, which is largely in agreement with the results reported for NtNAC29 in tobacco by Li et al. [18]. Subcellular localization and yeast autoactivation assays showed that SlNAC2-like is predominantly localized in the nucleus and possesses transcriptional activation activity, which matches the typical characteristics of NAC proteins reported in soybean by Shi et al. [19].

Transcription factors, particularly NAC family members, play critical roles in plant responses to high-temperature stress. In our expression pattern analysis, *SlNAC2-like* exhibited tissue-specific expression, and its expression was significantly induced by high temperature, similar to the characteristics of *RdNAC72* from *Rhododendron delavayi* [20]. Liu et al. [21] demonstrated that the NAC transcription factor *OsNTL3* positively regulates thermotolerance in rice. Liu et al. [22] reported that overexpression of *ZxNAC48* from *Zygophyllum xanthoxylum* in Arabidopsis enhanced plant thermotolerance, as evidenced by vigorous growth, increased stem biomass, improved photosynthetic capacity, and reduced membrane damage under high-temperature stress. In the present study, silencing *SlNAC2-like* in tomato reduced plant resistance to high temperature, suggesting that *SlNAC2-like* acts as a positive regulator of high-temperature tolerance in tomato. However, the molecular mechanism underlying its role in high-temperature tolerance remains unclear and warrants further investigation.

In this study, functional validation by VIGS was performed in the high-temperature-tolerant line Hm 2-2 rather than in both lines. Hm 2-2 was selected mainly because of its stronger high-temperature tolerance and the more pronounced phenotypic changes observed after gene silencing. Under high-temperature treatment, the difference in high-temperature sensitivity between *SlNAC2-like*-silenced and control plants was more readily distinguishable in this line, which facilitates the evaluation of loss-of-function phenotypes. Nevertheless, we acknowledge that the present functional evidence is based on a single genetic background. Whether *SlNAC2-like* plays a conserved role in other tomato lines, including the high-temperature-sensitive line BY 1-2 and other cultivars, remains to be determined. Functional redundancy among NAC family members and background-specific differences in hormone signaling and ROS metabolism could influence the phenotypic outcome of *SlNAC2-like* silencing. Therefore, future studies should validate the function of *SlNAC2-like* in additional genetic backgrounds, for example, by VIGS in BY 1-2 or by stable overexpression and CRISPR/Cas9 knockout in multiple tomato cultivars. In addition, a PDS positive control was not included in the VIGS experiment. Although the significant reduction in *SlNAC2-like* transcripts and the consistent phenotypic and physiological changes support the effectiveness of the silencing system, future studies should include a TRV::SlPDS positive control to further validate the VIGS system.

Under adverse environmental conditions such as biotic or abiotic stress, cells accumulate large amounts of ROS and free radicals [23]. Plants utilize antioxidant enzymes, including SOD, CAT, and POD, as well as MDA, to maintain ROS homeostasis and alleviate membrane lipid peroxidation [24]. Xi et al. [25] found that transgenic *Arabidopsis* overexpressing maize *ZmNAC074* exhibited significantly higher SOD and CAT activities and Pro content, and significantly lower MDA content, than controls under high-temperature conditions. Fang et al. [26] reported that overexpression of *SNAC3* in rice enhanced high-temperature tolerance. Transgenic plants overexpressing *SNAC3* showed significantly lower levels of H_2_O_2_, MDA, and relative electrolyte leakage than wild-type controls under high-temperature stress, suggesting that *SNAC3* confers stress tolerance by modulating ROS homeostasis. In our study, under high-temperature treatment, the activities of SOD, POD, and CAT and the Pro content in *SlNAC2-like*-silenced plants were significantly lower than those in controls, whereas the MDA content was higher, further indicating that *SlNAC2-like* is involved in tomato thermotolerance and is associated with ROS metabolism. Whether *SlNAC2-like* directly regulates ROS homeostasis remains to be determined. In this study, ROS accumulation was assessed by NBT and DAB staining, which provides a qualitative visualization of O_2_^−^ and H_2_O_2_ accumulation in situ. However, the staining intensity was not quantified, and therefore the differences in ROS accumulation between TRV::Empty and TRV::SlNAC2-like plants should be interpreted qualitatively. The increased oxidative stress in *SlNAC2-like*-silenced plants is nevertheless supported by the quantitative measurements of MDA content (Figure 12E) and the reduced activities of SOD, POD, and CAT (Figure 12A–C), which are consistent with impaired ROS scavenging. Future studies could quantify ROS levels using spectrophotometric or fluorometric assays to further validate these observations.

Plant responses to stress are often associated with hormone-mediated signal transduction pathways, and NAC transcription factors have been shown to participate in hormone-regulated signaling networks [27]. For instance, Meng et al. [13] found that *SlNAC72* is involved in the regulation of high-temperature stress in tomato by negatively regulating the expression of key JA biosynthesis genes (*SlLOX10*, *SlAOS1*, *SlAOC*). In our study, the expression of *SlNAC2-like* was induced by ABA, MeJA, and SA, suggesting its responsiveness to multiple hormonal cues. Under high-temperature conditions, silencing *SlNAC2-like* significantly reduced the transcript levels of ABA-related genes (*SlNCED1*, *SlNCED2*), SA-related genes (*SlTGA*, *SlNPR1*), and JA-related genes (*SlJAZ*, *SlLOXD*) compared with control plants. These results indicate that *SlNAC2-like* is associated with the normal expression of several key components of the ABA, SA, and JA signaling pathways under high-temperature stress. However, because these data are based on silencing rather than on direct binding assays, they support an association between *SlNAC2-like* and these pathways rather than direct transcriptional regulation. We therefore speculate that *SlNAC2-like* may contribute to tomato thermotolerance through its association with multiple hormone signaling pathways, possibly via crosstalk with ROS-scavenging systems. Nevertheless, the present data do not demonstrate direct transcriptional regulation of these hormone-related genes by *SlNAC2-like*. Identification of direct downstream target genes, for example, by ChIP-qPCR, EMSA, or dual-luciferase assays, as well as identification of interacting partners, will be required to elucidate the precise molecular mechanism.

## 4. Materials and Methods

### 4.1. Plant Materials

The tomato lines used in this study were the high-temperature-tolerant inbred line Hm 2-2 and the high-temperature-sensitive inbred line BY 1-2. The *Agrobacterium* strains harboring the VIGS vectors pTRV1 and pTRV2 were maintained in our laboratory. Seeds were sown in nursery pots filled with a commercial mixed substrate (peat∶perlite = 3∶1) (Xiangzheng Agriculture Technology, Liuyang, China) and grown in a growth chamber under a photoperiod of 14 h light (25–28 °C)/10 h dark (15–18 °C) with a relative humidity of 60–70%. Uniform six- to seven-leaf-old seedlings were subjected to different treatments, including high temperature (42 °C), ABA (0.1 mmol/L), SA (0.2 mmol/L), and MeJA (1.5 mmol/L). Each treatment consisted of 10 plants with three biological replicates. Leaf samples were collected at 0, 4, 12, 24, and 48 h after treatment, immediately frozen in liquid nitrogen, and stored at –80 °C until further use.

### 4.2. RNA Extraction and cDNA Synthesis

Total RNA was extracted from tomato leaves using the HiPure Plant RNA Mini Kit (Magen, Guangzhou, China). The extracted RNA was reverse-transcribed into cDNA using the StarScript Pro All-in-one RT Mix with gDNA Remover Kit (GenStar, Beijing, China), and the resulting cDNA was stored at –20 °C for further use.

### 4.3. Cloning of the Tomato SlNAC2-like Gene

Specific primers were designed using Primer Premier 5 software (PREMIER Biosoft International, Palo Alto, USA) (primer sequences are listed in Table 1). PCR amplification was performed using cDNA from leaves of the tomato line BY 1-2 as a template. The PCR products were separated by agarose gel electrophoresis, and the target bands were excised and purified using a gel extraction kit (GenStar, Beijing, China). The purified fragments were then ligated into the pMD19-T vector (Takara, Dalian, China). After transformation, positive clones were identified by colony PCR using universal primers (listed in Table 1), and the selected positive clones were sent to Sangon Biotech (Shanghai) Co., Ltd., Shanghai, China for sequencing.

### 4.4. Bioinformatics Analysis of Tomato SlNAC2-like

The ORF of the gene was predicted using the NCBI-ORF online tool, and conserved domain analysis was performed using the NCBI conserved domain search program. Physicochemical properties of the deduced protein were analyzed with the Expasy ProtParam tool, and hydrophilicity/hydrophobicity profiles were assessed using Expasy ProtScale. Secondary structure was predicted via SOPMA, and three-dimensional structural modeling was carried out using SWISS-MODEL. Signal peptide prediction was performed with SignalP 6.0, and transmembrane domain analysis was conducted using TMHMM-2.0. For phylogenetic analysis, the SlNAC2-like protein sequence and its homologues from 11 other plant species were retrieved from NCBI. Multiple sequence alignment was performed using DNAMAN 6.0 software (Lynnon Biosoft, Vaudreuil, QC, Canada) with default parameters. A phylogenetic tree was constructed using MEGA 6.0 software (Pennsylvania State University, University Park, PA, USA) with the Neighbor-Joining (NJ) method and the Poisson correction model. The reliability of the tree topology was evaluated by bootstrap analysis with 1000 replicates, and bootstrap values are shown at the corresponding nodes. Gaps were treated by pairwise deletion. The URLs of the online tools used are listed in Table 2.

### 4.5. qRT-PCR Analysis

qRT-PCR was performed using the 2 × RealStar Fast SYBR qPCR Mix (High ROX) kit (GenStar, Beijing, China) on a StepOne Real-Time PCR System (Applied Biosystems, Foster City, CA, USA). *SlACTIN* (Solyc03g078400) was used as the internal reference gene. For each biological replicate, leaf tissues from three randomly selected plants were pooled into one sample, and three technical replicates were performed for each sample. Three independent biological replicates were analyzed for each treatment. Relative gene expression levels were calculated using the 2^−ΔΔCt^ method [28]. The qRT-PCR primers used are listed in Table 1.

### 4.6. Subcellular Localization Analysis

Following the method described by Bi et al. [29], the coding sequence (CDS) of *SlNAC2-like* was cloned into the pCAMBIA1300-GFP vector via *Eco*RI and *Bam*HI restriction sites to generate the 35S∶SlNAC2-like-GFP recombinant construct. The recombinant plasmid was then transformed into *Agrobacterium tumefaciens* strain GV3101 competent cells (Weidi Biotechnology, Guangzhou, China). Subsequently, *Agrobacterium* cells harboring either the 35S∶SlNAC2-like-GFP recombinant construct or the empty 35S∶GFP vector were infiltrated into leaves of 4-week-old *Nicotiana benthamiana* plants. At 48 h post-infiltration, GFP fluorescence in the injected areas was observed using a confocal laser scanning microscope (C2-ER, Nikon, Tokyo, Japan). Three biological replicates were performed for each experiment. The primers used for construct generation (Sub-SlNAC2-like-F/R) are listed in Table 1.

### 4.7. Yeast Self-Activation Assay

Following the method of Chen et al. [30], the CDS of *SlNAC2-like* was cloned into the yeast pGBKT7 vector via *Eco*RI and *Bam*HI restriction sites. The recombinant plasmid pGBKT7-SlNAC2-like was transformed into yeast competent cells of strain AH109 (Weidi Biotechnology, Guangzhou, China). The transformed yeast cells were plated on SD/-Trp or SD/-His/-Trp/-Ade dropout media (Weidi Biotechnology, Guangzhou, China) and incubated at 30 °C for 3 d, after which α-galactosidase activity was assayed using X-α-gal as substrate. The co-transformation of pGADT7-T + pGBKT7-53 served as a positive control, while the pGBKT7 empty vector served as a negative control. All experiments were performed with three biological replicates. The primers used for vector construction (BD-SlNAC2-like-F/R) are listed in Table 1.

### 4.8. Functional Analysis of Tomato SlNAC2-like Using VIGS

A 212 bp fragment of *SlNAC2-like* (positions 715–926 of the CDS) was amplified with the specific primers VSlNAC2-like-F/R (Table 1) and inserted into the pTRV2 vector via *Eco*RI and *Bam*HI sites. The specificity of the fragment was evaluated by BLASTn (version 2.17.0) against the tomato genome. The only significant hits were three sequences representing the same *SlNAC2-like* gene in different cultivars or annotation versions (accession numbers NM_001278979.1, AK320303.1, and KC454000.1), all with 100% query coverage and 100% identity (E-value = 6e^−108^). No other tomato NAC family member showed significant sequence similarity, indicating a low risk of off-target silencing. The pTRV2 plasmid and the purified PCR product were digested with *Eco*R I and *Bam*H I at 37 °C for 2–4 h, inactivated at 85 °C for 10 min, and then ligated using T4 DNA ligase (Takara, Dalian, China)). The ligation product was transformed into *Escherichia coli* DH5α (Weidi Biotechnology, Guangzhou, China) by heat shock to generate the recombinant vector TRV∷SlNAC2-like. The empty pTRV2 vector (TRV∷Empty) and the recombinant vector (TRV∷SlNAC2-like) were separately transformed into *Agrobacterium tumefaciens* GV3101. *Agrobacterium* cultures harboring pTRV1 were mixed with those harboring TRV∷Empty or TRV∷SlNAC2-like at a 1∶1 (*v*/*v*) ratio, and the mixtures were infiltrated into leaves of tomato Hm 2-2 plants. Each treatment group consisted of 27 uniformly sized seedlings, and the experiment was performed as three independent biological replicates (9 seedlings per replicate per group). Two days after infiltration, the plants were transferred to a 42 °C incubator for high-temperature treatment. This time point was selected because pTRV-mediated silencing in tomato is typically established within 48 h after agroinfiltration, while the plants remain phenotypically normal before high-temperature treatment. Phenotypes were observed, photographed, and sampled at 2 d after treatment. The 2-day sampling time after high-temperature treatment was chosen because this is when the difference in wilting symptoms between TRV::Empty and TRV::SlNAC2-like plants became most evident and when the physiological and molecular changes were fully established. Silencing efficiency was confirmed by qRT-PCR at 2 days post-infiltration in all three biological replicates. For each replicate, leaf tissues from three randomly selected plants per group were pooled to form one sample, and the relative expression of *SlNAC2-like* was determined as described in Section 4.5.

### 4.9. Measurement of Morphological and Physiological Parameters

Biochemical assays: The activities of key antioxidant enzymes, including total SOD, POD, and CAT, as well as the contents of Pro and MDA, were determined using assay kits (Nanjing Jiancheng Bioengineering Institute, Nanjing, China) following the manufacturer’s instructions.

Histochemical staining: Accumulation of O_2_^–^ and H_2_O_2_ was detected by NBT and DAB staining, respectively, using assay kits (Wuhan Servicebio Technology Co., Ltd., Wuhan, China) according to the manufacturer’s protocols.

### 4.10. Statistical Analysis

All experimental data are presented as means ± standard error of the mean (SEM). A biological replicate was defined as an independent agroinfiltration batch (for VIGS experiments) or an independent treatment batch (for expression and hormone treatment experiments), as described in Section 4.1 and Section 4.8. Three biological replicates were performed for each experiment. Statistical analyses were performed using SPSS 20.0 software (IBM, Armonk, NY, USA). Differences among groups were assessed by one-way analysis of variance (ANOVA) followed by Tukey’s HSD test for multiple comparisons. *p* < 0.05 was considered statistically significant, and different lowercase letters in the figures indicate significant differences among groups. The specific comparisons performed for each dataset are described in the corresponding figure legends. Graphs were generated using GraphPad Prism 8.0 software (GraphPad Software, San Diego, CA, USA), and figure layouts were prepared using PowerPoint Open XML Presentation (Microsoft, Redmond, WA, USA).

## 5. Conclusions

In summary, a NAC transcription factor, *SlNAC2-like*, was cloned from tomato. The protein is an unstable hydrophilic protein, lacks a signal peptide and transmembrane domains, and is most closely related to SdNAC2-like from *Solanum dulcamara*. It is localized in the nucleus and possesses transcriptional activation activity. *SlNAC2-like* exhibits tissue-specific expression in tomato and is induced by high temperature, ABA, SA, and MeJA. Silencing this gene reduced plant resistance to high temperature, which was accompanied by decreased activities of SOD, POD, and CAT, reduced proline content, increased MDA content, and downregulated expression of hormone-signaling-related genes. These results suggest that *SlNAC2-like* may act as a positive regulator of tomato resistance to high temperature and that its role is associated with antioxidant enzyme activities and hormone-signaling-related genes. These changes should be interpreted as associations rather than evidence of direct regulation by *SlNAC2-like*. However, the present study does not elucidate the molecular mechanism by which *SlNAC2-like* regulates ROS homeostasis or hormone signaling, and whether it directly regulates these genes remains to be determined. This study provides a theoretical foundation for further investigation into the molecular mechanism by which *SlNAC2-like* regulates tomato responses to high-temperature stress. In the future, further experiments such as protein–protein interaction assays, identification of downstream target genes, and genetic transformation can be performed to elucidate the fine regulatory mechanism of *SlNAC2-like*.

## Figures and Tables

**Table 1:** Primers used in this study.

Primer	Sequence (5′–3′)	Application
SlNAC2-like-F	CTGTTTTCTACGTTTCAAC	Gene cloning
SlNAC2-like-R	TACAAATCATCGGCACGTG	
pMD19-F	CGCCAGGGTTTTCCCAGTCACGAC	Universal primer
pMD19-F/R	CGCCAGGGTTTTCCCAGTCACGAC	
SlACTIN-F	CTCTACATACTTGAGAGGTGCC	Reference gene
SlACTIN-R	AGACGAGGAGAAAACATCACAA	
qSlNAC2-like-F	CTTCACGTACTTTAATTCAC	Quantitative real-time PCR
qSlNAC2-like-R	ACATCTCATAACAACTCTGC	
qSlNCED1-F	CCCGATTTGGTATTCTGGATAAGTA	
qSlNCED1-R	GAGACGGATTTCGGATAAAACACT	
qSlNCED2-F	TGGTTTTCATGGGACATTCATTAGC	
qSlNCED2-R	ATCTCCCTTCTCAACTCCCTATTCC	
qSlNPR1-F	ACAAGTTGATGGCACGTCTG	
qSlNPR1-R	CCGATTCAAGTGCTCCTCTT	
qSlTGA-F	TACGGCAGGCTGATAACCTT	
qSlTGA-R	AGCTCAGTGCTCGAAGCCTA	
qLOXD-F	GGCTTGCTTTACTCCTGGTC	
qLOXD-R	AAATCAAAGCGCCAGTTCTT	
qSlJAZ-F	ATCAACCAGAGAAGGCAC	
qSlJAZ-R	TACCAAAACTCACACCAG	
Sub-SlNAC2-like-F	CCGGAATTCATGGTGGAATTGCAATTTCCTGCT	Vector construction
Sub-SlNAC2-like-R	CGCGGATCCAAACGGCTTTTGCAGATACATGAA	
BD-SlNAC2-like-F	ATGGCCATGGAGGCCGAATTCATGGTGGAATTGCAATTTCCTG	
BD-SlNAC2-like-R	CCGCTGCAGGTCGACGGATCCTCAAAACGGCTTTTGCAGATAC	
VSlNAC2-like-F/R	CCGGAATTCCTTCACGTACTTTAATTCAC	
VSlNAC2-like-F/R	CGCGGATCCACATCTCATAACAACTCTGC	

**Table 2:** Bioinformatics analysis websites.

Tools	Websites
NCBI-ORF	https://www.ncbi.nlm.nih.gov/orffinder/ (accessed on 25 February 2026)
NCBI conserved domain	https://www.ncbi.nlm.nih.gov/Structure/cdd/wrpsb.cgi (accessed on 25 February 2026)
Expasy ProtParam	https://web.expasy.org/protparam/ (accessed on 25 February 2026)
Expasy ProtScale	https://web.expasy.org/protscale/ (accessed on 25 February 2026)
SignalP 6.0	http://services.healthtech.dtu.dk/services/SignalP-6.0/ (accessed on 25 February 2026)
TMHMM-2.0	http://services.healthtech.dtu.dk/services/TMHMM-2.0/ (accessed on 25 February 2026)
SOPMA	http://npsa.lyon.inserm.fr/cgi-bin/npsa_automat.pl?page=/NPSA/npsa_sopma.html (accessed on 25 February 2026)
SWISS-MODEL	http://swissmodel.expasy.org (accessed on 25 February 2026)

## Data Availability

Data are contained within the article.

## Abbreviations

The following abbreviations are used in this manuscript:

ABA	Abscisic acid
ANOVA	One-way analysis of variance
CAT	Catalase
CDS	Coding sequence
DAB	3,3′-diaminobenzidine
GRAVY	Grand average of hydropathicity
H_2_O_2_	Hydrogen peroxide
JA	Jasmonic acid
MDA	Malondialdehyde
MeJA	Methyl jasmonate
NBT	Nitroblue tetrazolium
O_2_^–^	Superoxide anion
ORF	Open reading frame
POD	Peroxidase
Pro	Proline
qRT-PCR	Quantitative real-time PCR
ROS	Reactive oxygen species
RT-PCR	Reverse transcription PCR
SA	Salicylic acid
SEM	Standard error of the mean
SOD	Superoxide dismutase
VIGS	Virus-induced gene silencing
